# Pulmonary Hypoplasia Associated with Congenital Heart Diseases: A Fetal Study

**DOI:** 10.1371/journal.pone.0093557

**Published:** 2014-04-03

**Authors:** Isabelle Ruchonnet-Metrailler, Bettina Bessieres, Damien Bonnet, Shamila Vibhushan, Christophe Delacourt

**Affiliations:** 1 AP-HP, Hôpital Necker-Enfants Malades, Service de Pneumologie Pédiatrique, Centre de Référence pour les Maladies Respiratoires Rares de l’Enfant, Paris, France; 2 AP-HP, Hôpital Necker-Enfants Malades, Service Histo-Embryologie et Cytogénétique, Paris, France; 3 INSERM UMR 781, Paris, France; 4 AP-HP, Hôpital Necker-Enfants Malades, Cardiologie Pédiatrique, M3C-Centre de Référence pour les Malformations Cardiaques Congénitales Complexes Paris, France; 5 Université Paris-Descartes, Paris, France; 6 INSERM, U955, IMRB, Equipe 04, Créteil, France; University of Southern California, United States of America

## Abstract

**Background:**

Abnormalities of the fetal pulmonary vasculature may affect lung morphogenesis. Postnatal studies have suggested that pulmonary hypoplasia (PH) may be associated with congenital heart diseases (CHDs).

**Objective:**

To determine the prevalence of PH associated with CHDs, and to evaluate whether CHDs with right outflow obstruction were associated with the highest risk of lung growth impairment.

**Methods:**

Between January 2006 and December 2010, fetuses with CHD obtained following the termination of pregnancies due to fetal abnormalities were examined in a prospective manner for the detection of heart and lung defects. CHDs were classified into five pathophysiological groups. Lung weight (LW), body weight (BW), and LW/BW ratio were analyzed for each case. The expression of CD31 and VEGF in the lung was evaluated by immunohistochemistry.

**Results:**

Fetuses with CHDs and right outflow obstruction had significantly lower LW for a given BW, and significantly lower LW/BW ratios for a given gestational age. When defining PH as a fetal LW/BW ratio <0.015 before 28 weeks, and <0.012 after 28 weeks, PH was detected in 15 of the 119 fetuses analyzed (13%). It was significantly associated with CHD with right outflow obstruction, independently of chromosomal abnormalities and associated extracardiac abnormalities (*p*<0.03). Right outflow obstruction was detected in 60% of the fetuses with CHD and PH, but in only 32% of those with CHD but no PH. In fetuses with right outflow obstruction, no difference was observed between those with PH and those without PH, in terms of the ratio of pulmonary artery diameter to aortic diameter, lung CD31 expression, or lung VEGF expression.

**Conclusion:**

CHDs with right outflow obstruction are a significant risk factor for prenatally acquired PH. The occurrence of fetal PH is not correlated with abnormalities of the pulmonary vasculature, suggesting the involvement of perfusion-independent mechanisms.

## Introduction

Lung morphogenesis is a complex process, subject to tight temporal and spatial regulation by epithelium-mesenchyme interactions, and resulting in a functional air-blood interface for gas exchange at birth [1], [2]. There is also growing experimental evidence implicating the underlying vasculature in tissue patterning [3], [4], [5]. The early disruption of pulmonary vascular growth alters the branching morphogenesis of the airways [3], [4], and leads to a defect in primary septae formation during distal lung morphogenesis [5]. Decreases in pulmonary blood flow at a later stage of gestation significantly alter alveolarization [6]. Complete interruption of the pulmonary circulation is associated with pulmonary hypoplasia (PH) [7]. However, much less is known about interactions between the growing fetal pulmonary vasculature and organogenesis in humans. If such interactions do occur, they may be compromised in the presence of congenital heart disease (CHD). Some postnatal autopsy studies have reported a low density of intrapulmonary vessels [8], [9], [10], and a reduction in the number of alveoli [9], [10] in fetuses with congenital pulmonary atresia or tetralogy of Fallot. In children or adults living with CHDs, PH is suggested by a persistently low lung volume and lung diffusing capacity [11], [12], [13], [14]. However, these previous studies were based on postnatal recruitment, making it impossible to assert the existence of a causal link between CHD and impaired fetal lung development. The physiology of the pulmonary circulation changes considerably at birth, so postnatal evidence of altered lung development may reflect these new physiological conditions, rather than the disruption of lung morphogenesis *in utero*. Moreover, these postnatal studies consider many environmental factors that may themselves interfere with lung growth. Fetal studies are therefore required to determine the direct consequences of CHDs in terms of lung growth.

Most CHDs are now diagnosed prenatally [15]. The most complex defects, or associations of CHDs with other abnormalities, may lead to the termination of pregnancy for fetal abnormality (TOPFA) [15]. Taking advantage of a prospective collection of TOPFA with CHD at our center since 2006, including a complete description of the heart defects and of lung development, we investigated the prevalence of PH associated with CHDs, and evaluated whether CHDs with right outflow obstruction were associated with the highest risk of lung growth impairment.

## Methods

### Data Collection

This study was carried out at Necker Hospital, a referral center for prenatal and postnatal CHD care. Most severe defects identified prenatally lead to a termination of pregnancy at the request of the parents. According to French law, TOPFA may be carried out at any term during pregnancy. The fetuses aborted in TOPFA procedures are routinely examined in a standardized manner, to allow the full description of defects and to make it possible to provide informative genetic counseling. Written parental consent for such studies is obtained systematically. From January 2006 to December 2010, fetuses with CHD aborted in a TOPFA procedure were examined prospectively, in a standardized manner, for heart and lung defects. A complete anatomical description of the CHD was obtained and lung weight was systematically measured. We reviewed the files of the fetuses evaluated during this period. We obtained the following information from reports: gestational age at diagnosis, gestational age at TOPFA, biometric parameters, placenta appearance and measurements, anatomic description of the cardiac malformation, lung weight, associated malformations, associated chromosomal aberrations. When available, the diameters of the aorta (Ao) and pulmonary artery (PA) were also noted. We calculated the ratio of the diameter of the PA to the diameter of the ascending Ao (PA/Ao ratio). This ratio remains fairly constant during gestation, at about 1.2 [16], [17]. This study was approved by the Institutional Review Board of the French Society for Respiratory Medicine (CEPRO 2013–021). All samples and records were anonymized.

PH was evaluated by different approaches. Raw data were first analyzed. Individual lung weight (LW) was plotted versus body weight (BW). The regression model providing the highest correlation coefficient was run. This model permitted the calculation for each case of a predicted LW and of the residual, which corresponds to the deviation of the observation from the sample mean. PH was also defined according to previously established definitions. Fetal PH is usually defined as a fetal LW-to- BW ratio <0.015 before 28 weeks, and <0.012 after 28 weeks of gestation [18]. As this criterion may be too stringent for the detection of mild PH, we also expressed the observed LW/BW values as a percentage of the expected value estimated from the following equation: expected LW/BW = 0.088–0.044×log(weeks of gestation) [18]. CHDs were coded with the International Pediatric and Congenital Cardiac Code (IPCCC), and initially classified into five different pathological groups, derived from the most recent recommendations [15]: abnormalities of right ventricular outflow, including pulmonary stenosis, tetralogy of Fallot (TOF), and pulmonary atresia with ventricular septal defects; abnormalities of left ventricular outflow; transposition of great arteries and variants; abnormalities of venous return, including all abnormalities of the venous pole of the heart; other CHDs, including complex CHD with abnormalities of both right and left outflow.

### Immunostaining

Pieces of lung tissue were fixed in formalin and embedded in paraffin. Sections (5 μm thick) were cut, dehydrated in xylene and then incubated in a graded series of ethanol solutions.

Immunohistochemistry for CD31 was performed with mouse anti-human, PECAM-1/CD31 (diluted 1/50; MO823; Dako, Glostrup, Denmark) or mouse monoclonal anti-VEGF (diluted 1/400; catalog number ab 1316, Abcam, Cambridge, UK) antibodies, according to the manufacturer’s instructions. The lung sections used as negative controls were treated with the same dilution of non immune mouse IgG1 (cat number 550878, BD Pharmingen, CA). The slides were incubated with horseradish peroxidase (HRP)-conjugated streptavidin and counterstained with hematoxylin. The tissue sections were dehydrated and mounted on slides for examination under a light microscope. Photomicrographs were obtained with a digital camera.

CD31-expressing cells were quantified by the point counting method, which is based on the principles of Weibel [19]. Quantification was carried out on light microscopy fields, at an overall magnification of ×40, with a 42-point, 21-line eyepiece graticule placed on the television screen. We evaluated five fields per slide, by a systematic sampling method beginning at a random starting point. The percentage of CD31-positive cells is expressed as the number of points overlying these cells as a percentage of the total number of points counted.

### Statistical Analysis

Quantitative variables are presented as medians and interquartile ranges (IQR), corresponding to the difference between the third (75% of the distribution) and first (25% of the distribution) quartiles. Groups were compared in nonparametric Mann-Whitney or Kruskal-Wallis tests. Qualitative variables are expressed as percentages and were compared in Chi^2^ tests. All variables significant at the 5% level were entered into a multivariate logistic regression model, together with variables known to have an impact on lung growth. Data were analyzed with XLSTAT software, and values of *p*<0.05 were considered significant.

## Results

We analyzed prospectively 125 fetuses with CHD. Six fetuses were excluded due to an association of congenital diaphragmatic hernia and macroscopic ipsilateral PH. Overall, 70 (59%) of the 119 fetuses analyzed were male. Median term (IQR) at TOPFA was 27 (23–32) weeks. The cohort was split into five groups on the basis of the CHD: right outflow obstruction (*n* = 42, 35%), left outflow obstruction (*n* = 46, 39%), transposition of great arteries (*n* = 9, 8%), abnormal pulmonary venous return (*n* = 3, 2%), and other CHDs, including complex CHD (*n* = 19, 16%). Details of the CHD diagnoses are reported Table 1. As only a small number of fetuses presented an isolated pulmonary venous return abnormality, we therefore grouped these cases with the “other” group for the analysis. Eighty fetuses (67%) had extracardiac abnormalities, including facial dysmorphism, growth abnormalities or the involvement of other organs. In 23 of these fetuses, at least one of the abnormalities detected was considered to have a potentially direct effect on lung growth: intrauterine growth retardation (IUGR) (*n* = 13), renal hypoplasia (*n* = 9), omphalocele (*n* = 4), thoracic dystrophy (*n* = 1), hydrothorax (*n* = 1). Karyotyping was performed for 106 fetuses and was abnormal in 17(16%).

**Table 1:** CHD diagnosis (*n* = 119).

Diagnosis	*n*	IPCCC codes
Right outflow obstruction:		
Tetralogy of Fallot,	9	01.01.01
Pulmonary atresia+ventricular septal defect	13	01.01.06
Pulmonary atresia+tricuspid atresia	3	09.05.11; 06.01.01
Tricuspid atresia	2	06.01.01
Tricuspid atresia+pulmonary stenosis	1	06.01.01; 09.10.01
Ebstein’s malformation	1	12.02.77
Pulmonary stenosis	2	09.05.92
Pulmonary atresia+intact ventricular septum	3	01.01.07
Right cardiomegaly	4	10.17.18
Right ventricular hypoplasia,	2	07.02.00
Tricuspid atresia+right ventricular hypoplasia+pulmonary arterial hypoplasia	1	06.01.01; 07.02.00; 09.10.11
Pulmonary arterial hypoplasia	1	09.10.11
Left outflow obstruction:		
Left ventricular hypoplasia	8	07.07.00
Left ventricular hypoplasia+mitral atresia	2	07.07.00; 06.02.01
Left ventricular hypoplasia+aortic atresia	2	07.07.00; 09.15.03
Left ventricular hypoplasia+mitral atresia+aortic atresia	11	07.07.00; 06.02.01; 09.15.03
Left ventricular hypoplasia+anomalous pulmonary venous connection	2	07.07.00; 04.08.07
Aortic coarctation	4	09.29.01
Aortic arch hypoplasia	6	09.29.11
Interrupted aortic arch	2	09.29.31
Mitral valvar abnormality	5	06.02.00
Aortic valvar abnormality	3	09.15.19
Mitral atresia+Aortic atresia	1	06.02.01; 09.15.03
Transposition of great arteries and variants:		
Transposition of great arteries	2	01.05.01
Transposition of great arteries & intact ventricular septum	1	01.01.02
Congenitally corrected transposition of great arteries	2	01.01.03
Double outlet right ventricle: transposition type	2	01.01.18
Transposition of great arteries+pulmonary trunk hypoplasia	2	01.05.01; 09.07.11
Anomalous pulmonary venous connection	3	04.08.07
Other CHDs – complex CHDs:		
Ventricular septal defect	5	07.10.00
Abnormalities of systemic venous return	2	04.00.07
Common arterial trunk (truncusarteriosus)	2	09.01.01
Atrioventricular septal defect	4	06.06.00
Atrial septal defect	2	05.03.00
Double outlet right ventricle	1	01.05.03
Aortic arch branch abnormality	1	09.30.00
Left ventricular hypoplasia+aortic arch hypoplasia + pulmonary atresia	1	07.07.00; 09.29.11; 09.05.11
Heterotaxy	1	01.01.22, 03.01.02, 01.01.04, 06.06.00

**Figure 1:**
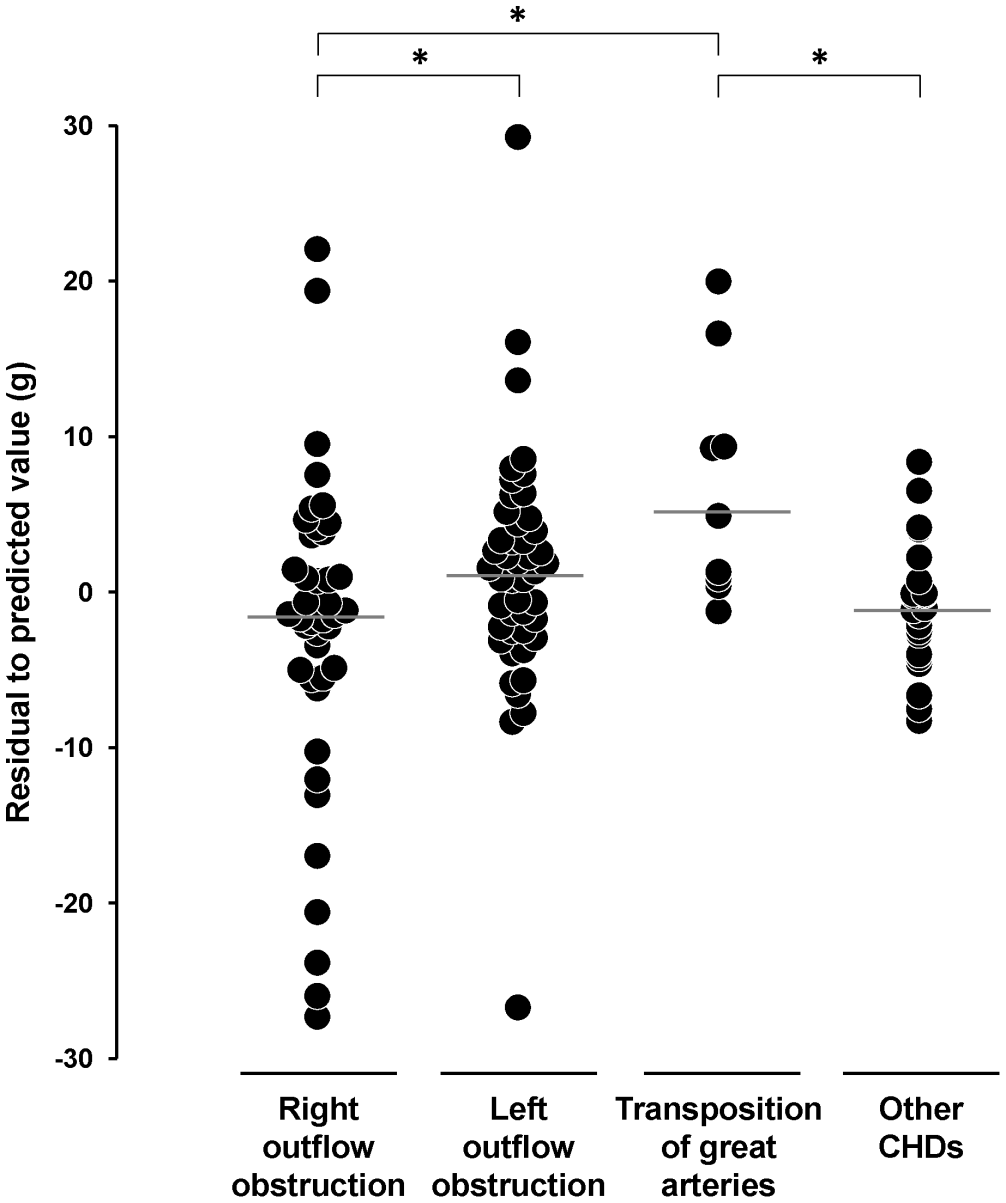
Individual residual values. Fetuses are classified into four categories on the basis of the CHD. Values are deviations of each observation from the sample mean. Horizontal bar: median value in each subgroup. Kruskal-Wallis test: *p* = 0.008. *p<0.05 for the comparison between subgroups.

Raw data were first analyzed (Table 2). We plotted LW vs BW for each case in our cohort, The regression model providing the highest correlation coefficient between LW and BW was a degree 2 polynomial function (Figure S1; R∧2 = 0.718). Equation was: LW = 1.585+0.023*BW - 2E-6*BW∧2, LW and BW being expressed in grams. From this equation, LW values were expressed as percentage of the sample mean, and residuals were calculated. Fetuses with CHD and right outflow obstruction had significantly lower LW values, expressed as % of sample mean, and significantly lower residuals than fetuses with left outflow obstruction or with transposition of great arteries (Figure 1).

**Table 2:** Characteristics of the fetuses, assigned to four subgroups according to the category of CHD.

	Right outflow obstruction(n = 42)	Left outflow obstruction(n = 46)	TGA (n = 9)	Other CHDs (n = 22)
**Male/Female**	25/17	26/20	7/2	12/10
**Gestational age (wks)**	28.0 (24.0–31.0)	26.5 (23.0–32.0)	27.0 (24.0–33.0)	26.0 (23.3–31.8)
**BW (g)**	1050 (393–1710)	950 (473–1623)	900 (720–1850)	895 (407–1825)
**LW (g)**	23.3 (6.6–30.3)	21.8(13.3–29.6)	25.0 (18.0–45.0)	17.9 (9.7–31.5)
**LW (% of sample mean)§** *	90.8 (59.1–113.0)	112.0 (84.5–124.1)#	124.5 (105.6–129.8)#	97.0 (71.8–102.4)
**Residual (g)§** *	−1.4 (−5.4–3.1)	1.5 (−2.4–4.3)#	4.9 (0.9–9.4)#	−1.1 (−3.7–0.5)
**LW/BW** *	0.021 (0.014–0.026)	0.024 (0.018–0.029)#	0.025 (0.024–0.025)	0.019 (0.015–0.023)
**LW/BW (% of expected)** ¶ *	80.6 (56.7–101.7)	97.1 (76.9–109–0)#	105.3 (91.7–114.8)#	84.2 (66.0–93.3)
**Chromosomal abnormality (%)**	16.2	11.6	0	33.3
**Any associated extracardiac abnormality**	16.7	19.6	0	31.8

Values are medians (IQR) or *n* (% of each subgroup). BW: body weight; LW: lung weight. § The mean values for the population and the residuals are derived from the regression model relating LW and BW (see text and Figure S1);

¶ expected values derived from [18];

*p value<0.05 in multiple group analysis;

#*p* value <0.05 when comparing each subgroup with the one with right outflow obstruction.

Very similar results were obtained when using in our cases the pre-defined equation for predicted values of LW/BW ratio. Significantly lower LW/BW values, expressed as a percentage of the expected value, were measured in fetuses with right outflow obstruction than in those from other subgroups (*p*<0.02) (Figure S2). However, these analysis with continuous data demonstrated a large overlap in LW or LW/BW values between CHD subgroups, indicating that CHDs with right outflow obstruction were not consistently associated with impaired lung growth.

Finally, PH was analyzed, using the pre-defined cut-offs of fetal LW/BW ratio <0.015 before 28 weeks, and <0.012 after 28 weeks of gestation. With these cut-offs, 15 fetuses (13%) had PH, the characteristics of which are presented in Table 3. Sex ratio, gestational age, body weight and the presence or absence of an associated extracardiac abnormality did not differ between fetuses with and without PH (Table 4). By contrast, PH was significantly associated with a higher frequency of abnormal karyotypes (*p*<0.02), and a higher rate of CHD with right outflow obstruction (*p*<0.04). The percentage of fetuses with right outflow obstruction in the group with PH was twice that in the group without PH: 60% versus 32%, respectively. Univariate and multivariate analysis are presented Table 5. CHD with right outflow obstruction was identified as a significant risk factor for PH, independently of chromosomal abnormality and associated extracardiac abnormalities (*p*<0.03).

**Table 3:** Characteristics of fetuses with CHD and PH.

	Sex	Cardiac malformation	Gestational age (wks)	BW (g)	LW/BW	Associated malformations	Karyotype
1	M	Valvular mitral dysplasia	35	3130	0.007	None	Normal
2	M	Tricuspid atresia+mitral atresia,	36	3000	0.009	None	Normal
3	M	Mitral atresia+aortic atresia	15	85	0.005	Renal fusion, campylodactyly	Mosaic T18
4	F	Pulmonary atresia+Tricuspid atresia	27	860	0.007	None	Normal
5	M	Tetralogy of Fallot	22	266	0.005	Renal hypoplasia, VLBW, thymic hypoplasia	Duplication 19p13
6	F	Pulmonary atresia with septal defect	24	240	0.005	Duodenal stenosis, Campylodactyly	46XXX
7	F	Interrupted aortic arch	23	210	0.011	IUGR, Campylodactylia	Triplody
8	M	Pulmonary atresia with septal defect	29	1380	0.002	Hexadactyly	Not available
9	M	Right cardiomegaly	30	2250	0.011	None	Normal
10	F	Pulmonary atresia with septal defect	22	259	0.009	Renal hypoplasia	T14
11	M	Aortic coarctation	16	88	0.013	Renal cystic dilatation	Not available
12	M	Atrial septal defect	25	580	0.013	Clynodactyly, omphalocele	Normal
13	F	Right ventricular hypoplasia and septal defect	18	210	0.010	None	Normal
14	M	Tetralogy of Fallot	29	1560	0.005	None	Normal
15	M	Right cardiomegaly	33	2800	0.007	Hydrothorax	Normal

**Table 4:** Characteristics of the fetuses, assigned to two subgroups according to the association of PH with CHD.

	CHD and no PH (*n* = 104)	CHD and PH (*n* = 15)
Male	60 (58)	10 (67)
Gestational age (wks)	27.0 (24.0–32.0)	25.0 (22.0–29.5)
BW (g)	965 (557–1715)	580 (225–1905)
LW (g)	23.55 (13.35–32.90)	2.50 (1.65–13.64)**
LW/BW	0.023 (0.019–0.028)	0.007 (0.005–0.010)**
LW/BW (% of expected)	93.1 (77.3–107.8)	30.7 (20.1–38.1)**
Chromosomal abnormality	12/93 (13)	5/13 (38)*
Any associated extracardiac abnormality	68 (65)	12 (80)
IUGR	11 (11)	2 (13)
Renal hypoplasia	7 (7)	2 (13)
Omphalocele	3 (3)	1 (7)
Thoracic dystrophy	1 (1)	0 (0)
Hydrothorax	0 (0)	1 (7)
Right outflow obstruction	33 (32)	9 (60)*
Left outflow obstruction	42 (40)	4 (27)
TGA	9 (9)	0 (0)
Other CHDs	20 (19)	2 (13)

Values are medians (IQR) or *n* (% of each subgroup). BW: body weight; LW: lung weight.

*P value<0.05;

**P value<0.0001.

**Table 5:** Univariate and multivariate analyses assessing the risk of PH as a function of CHD category, presence of an abnormal karyotype and the presence of a malformation likely to interfere with lung growth (IUGR, renal hypoplasia, omphalocele, thoracic dystrophy, or hydrothorax).

Variable	OR	95% CI	p value
***Univariate analysis***			
CHD with right outflow obstruction	3.227	1.061–9.817	0.039
Abnormal karyotype	4.219	1.183–15.041	0.026
Associated extracardiac abnormality	2.389	0.728–7.834	0.151
***Multivariate analysis***			
CHD with right outflow obstruction	3.812	1.094–13.281	0.036
+ Abnormal karyotype	4.619	1.218–17.507	0.024
CHD with right outflow obstruction	4.353	1.193–15.875	0.026
+ Abnormal karyotype	3.737	0.941–14.848	0.061
+ Associated extracardiac abnormality	2.716	0.684–10.787	0.156

**Figure 2:**
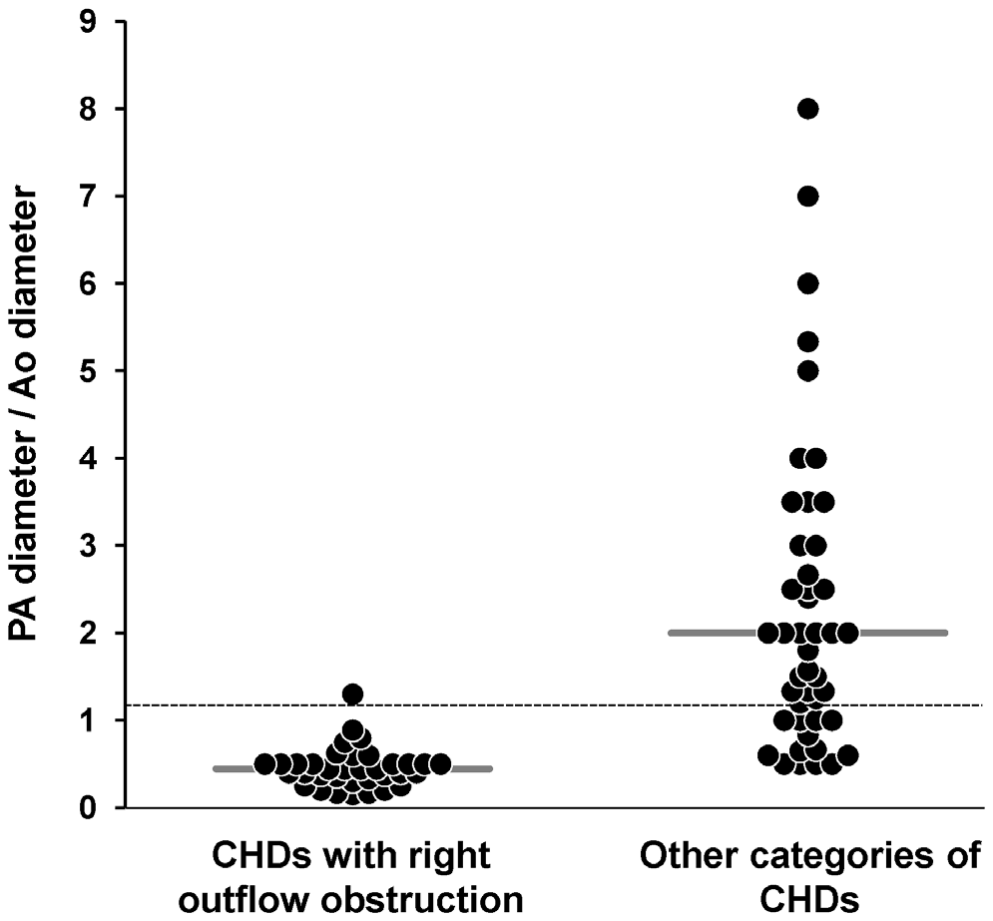
Individual PA/Ao values. Values were available for 35 fetuses with CHD and right outflow obstruction, and 45 fetuses with CHDs of other types. Horizontal bar: median value in each subgroup. Dashed horizontal line: expected normal AP/Ao value (i.e. 1.2). *p*<0.0001 for comparisons between subgroups.

**Figure 3:**
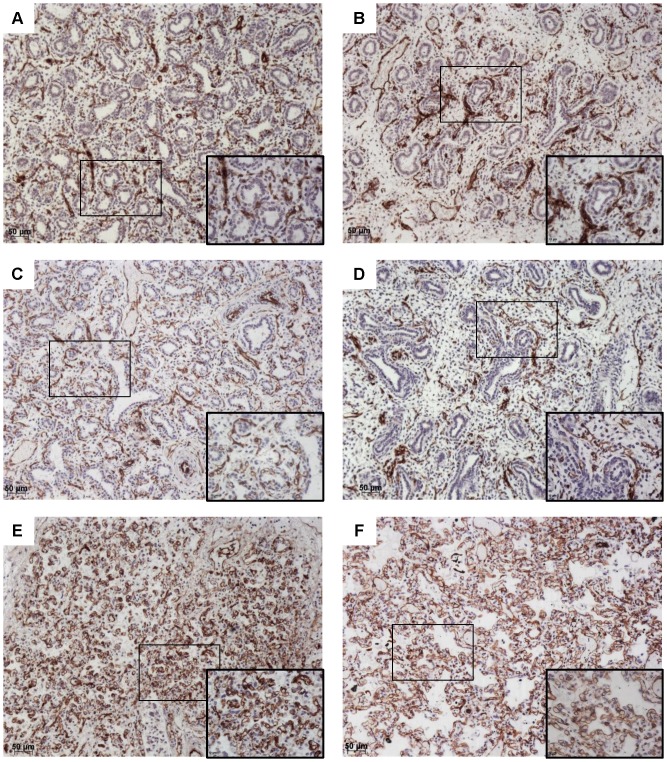
CD31 immunohistochemistry. Original magnification×10, and ×40 magnification of the area identified by a rectangle. CD31 (brown) and counterstaining with hematoxylin. Fetuses with PH (A, C, E) were compared with fetuses of a similar gestational age without PH (B, D, F). A: Fetus with right ventricular hypoplasia and a septal defect, 18 weeks, LW/BW = 0.010; B: Fetus with pulmonary atresia and a septal defect, 16 weeks, LW/BW = 0.024; C: Fetus with tetralogy of Fallot, 22 weeks, LW/BW = 0.005; D: Fetus with an atrioventricular septal defect, 17 weeks, LW/BW = 0.027; E: Fetus with pulmonary atresia and tricuspid atresia, 36 weeks, LW/BW = 0.009; F: Fetus with tetralogy of Fallot, 33 weeks, LW/BW = 0.029.

**Figure 4:**
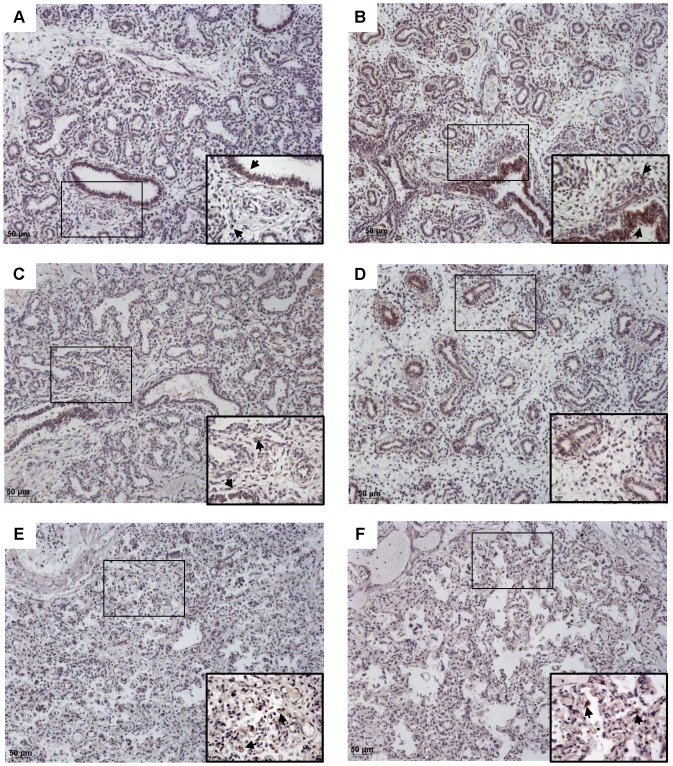
VEGF immunohistochemistry. Original magnification×10, and ×40 magnification of the area identified by a rectangle. VEGF (brown) and counterstaining with hematoxylin. Fetuses with PH (A, C, E) were compared withfetusesof a similar gestational age without PH (B, D, F). A: Fetus with right ventricular hypoplasia and a septal defect, 18 weeks, LW/BW = 0.010; B: Fetus with pulmonary atresia and a septal defect, 16 weeks, LW/BW = 0.024; C: Fetus with tetralogy of Fallot, 22 weeks, LW/BW = 0.005; D: Fetus with atrioventricular septal defect, 17 weeks, LW/BW = 0.027; E: Fetus with pulmonary atresia and tricuspid atresia, 36 weeks, LW/BW = 0.009; F: Fetus with tetralogy of Fallot, 33 weeks, LW/BW = 0.029.

We investigated whether the significant association between right outflow obstruction and PH was related to abnormalities of the pulmonary vascular bed. The diameters of the ascending aorta (Ao) and main pulmonary artery (PA) were available for 80 fetuses with CHDs, including 35 cases of CHD and right outflow obstruction. As expected, the PA/Ao ratio was significantly lower in the group of fetuses with CHD and right outflow obstruction, attesting for reduced PA diameters (Figure 2, *p*<0.0001). However, there was no correlation with PH in this subgroup. The median value of this ratio (IQR) was 0.50 (0.43–0.65) in fetuses with right outflow obstruction and PH (*n* = 9), versus 0.40 (0.37–0.50) in those without PH (*n* = 26). Similarly, we found no correlation between PA/Ao ratio and LW/BW value, expressed as a percentage of the predicted value (*r* = 0.06, *p*>0.7). We investigated the peripheral pulmonary vasculature in fetuses with right outflow obstruction by immunohistochemistry and CD31 labeling (Figure 3). PH was not associated with a low level of lung CD31 labeling. The median values of CD31-expressing cell density obtained by the point-counting method were 0.21 (0.19–0.26) for fetuses without PH, and 0.26 (0.18–0.38) for those with PH. We also analyzed expression of the key angiogenic factor VEGF (Figure 4). In the fetuses with the lowest gestational ages, VEGF was present in the branching airway epithelium and mesenchymal cells (Fig. 4 A–B). In the oldest fetuses (greatest gestational age), VEGF labeling was scattered, but was observed particularly in the distal epithelium and mesenchyme (Fig. 4 E–F). No reliable quantification was possible. No obvious difference in the pattern of VEGF expression was noted between fetuses with and without PHPH.

## Discussion

The interactions between the pulmonary vasculature and early lung morphogenesis in humans are still poorly understood. Concomitant alterations of the pulmonary vascular bed and alveolarization have been described on the basis of autopsies carried out on children with CHD and right outflow obstruction [9], [10]. The results of these studies suggest that hemodynamic factors (i.e. altered pulmonary blood flow), or anatomical factors (i.e. a change in the number and size of the pulmonary vessels), or both, may interfere with lung growth, at least postnatally. However, the hemodynamic changes associated with CHDs in the fetus differ considerably from those occurring postnatally [20], and the current lack of fetal data make it impossible to determine whether lung parenchymal and vascular abnormalities in CHDs have a common developmental origin, early in the course of morphogenesis, or whether they occur more gradually, after birth. This study is the first to describe a series of fetuses with CHD and the parallel evaluation of lung development. The principal strengths of this study are the large number of cases analyzed, the standardized prospective collection of data, and the wide range of gestational ages. Our findings confirm that right outflow obstruction is a significant risk factor for PH. Moreover, when PH occurred in cases with right outflow obstruction, it does not seem to be associated with a decrease in the size of the pulmonary vascular bed, suggesting perfusion-independent mechanisms.

Fetal PH is usually assessed by calculating the LW/BW ratio. This simple way of defining PH has been validated by the demonstration of a close correlation between LW/BW ratio and lung DNA content for gestation [18]. The lower limit of the normal range for this ratio has been estimated at 0.012 for fetuses of at least 28 weeks of gestation, and 0.015 for fetuses of lower gestational ages [18]. These thresholds are useful for the identification of severe PH, generally occurring secondary to defects such as congenital diaphragmatic hernia, renal abnormalities with severe oligohydramnios and musculoskeletal abnormalities [21]. Our findings identify CHD with right outflow obstruction as a significant risk factor for severe PH, even after adjustment for extracardiac malformations and chromosomal aberrations. All fetuses with these criteria for PH had LW/BW values below 50% of the expected value. Our findings also suggest that CHDs with right outflow obstruction may be associated with less severe PH. In this group of fetuses, individual LW values for a given BW, and individual LW/BW values for a given gestational age differed significantly from those for fetuses with CHDs with left outflow obstruction, or with transposition of the great arteries. Both these categories of defect are thought to have an effect on the fetal pulmonary vascular bed opposite to that for CHDs with right outflow obstruction. The number of peripheral arteries is normal or high in CHDs with left outflow obstruction [8], whereas it is low in CHDs with right outflow obstruction [8]. In terms of hemodynamics, transposition of the great arteries has been shown to increase fetal pulmonary blood flow [20]. We also observed some degree of PH in a subgroup of fetuses with mixed CHDs, with an unpredicted impact on the fetal pulmonary vascular bed. Given the physiological complexity of these CHDs, we can assume that they display some of the alterations to the fetal pulmonary vasculature physiology reported for CHDs with right outflow obstruction.

An association between PH and CHDs with right outflow obstruction has previously been suggested purely on the basis of postnatal studies. Autopsy studies have shown affected infants to have an abnormally small number of alveoli [9], [10]. Based on this parameter, the prevalence of PH appears to be higher postnatally than in our series of fetuses: almost half the children with right outflow obstruction have less than 50% the expected number of alveoli, and two thirds have less than 75% the expected number [10]. The impaired alveolarization in patients with right outflow obstruction may be an important cause of the low lung volumes measured in children and adults operated for the tetralogy of Fallot [12], [13], [22], [23], [24], or for other CHDs with right outflow obstruction [25], [26]. Interestingly, our findings are consistent with previous data suggesting that CHDs without right outflow obstruction are not associated with small lung volumes [27], [28]. In these postnatal studies, it was assumed that PH was directly linked to the decrease in postnatal pulmonary blood flow. However, our fetal study suggests that the mechanisms responsible for PH may be less clearcut. PH may already have been acquired during the fetal period, without correlation with the size of pulmonary arteries or the density of intrapulmonary vessels, suggesting a role for perfusion-independent mechanisms.

Fetuses with right outflow obstruction had low AP/Ao ratios, consistent with a small diameter of the pulmonary arteries, but no correlation was found in this subgroup between the degree of decrease in the AP/Ao ratio and the occurrence of PH. Similarly, no decrease in CD31-positive cell density was observed in fetuses with right outflow obstruction and PH. We also investigated the expression of the key angiogenic factor VEGF. In control lungs, VEGF is known to be expressed by mesenchyme and epithelium throughout lung development [29], [30]. Our findings are consistent with previous reports that VEGF levels in the lung decrease with gestational age [31], with this factor preferentially localized in peripheral airway epithelial cells in the last three months of gestation [3]. The role of VEGF in pulmonary angiogenesis has been clearly demonstrated. VEGF blockade during early stages of lung development leads to a progressive decrease in microvascular density [4]. Conversely, the overproduction of VEGF in the developing respiratory epithelium stimulates the growth of the pulmonary blood vessels [32]. In our study, VEGF levels were found to be normal in the hypoplasic lungs. Similarly, no decrease in VEGF levels in fetal lungs was reported in a previous study, in cases of PH secondary to congenital diaphragmatic hernia or renal abnormalities [31]. Overall, our findings suggest that there is no direct causal relationship between a smaller pulmonary vascular bed associated with right outflow obstruction, and the occurrence of PH during the fetal period. Similarly, Haworth *et al*. demonstrated, in eight newborns that died within the first days of life, that the small diameter of the pulmonary arteries associated with pulmonary atresia was not accompanied by a decrease in the number of alveoli [8]. The extent of postnatal alveolar loss seems to be related to the decrease in pulmonary blood flow rather than the number or size of the pulmonary arteries, as patients with higher levels of pulmonary blood flow due to the presence of large systemic collateral arteries or large surgical shunts seemed to have a larger number of alveoli, despite having a smaller number of arteries or arteries with a smaller diameter [10]. Levels of spontaneous blood flow in the fetal pulmonary circulation is low, due to the high level of pulmonary vascular resistance [20], and it is therefore possible that the changes induced by CHDs with right outflow obstruction have little impact during the prenatal period. This hypothesis is supported by the recent demonstration that postnatal decreases in pulmonary blood flow induce a lung remodeling process, including downregulation of the expression of key controllers of alveolar multiplication, such as metalloproteinase (MMP)-2 and VEGF [33]. Thus, the main explanation for our findings may be based on altered interactions between endothelial cells and the surrounding mesenchyme and epithelium. Developing blood vessels have been shown to influence the spatial distribution of key molecular mediators of branching [4]. Endothelial cell dysfunction in fetuses with CHDs may therefore contribute to PH, rather than reduced pulmonary flow *per se*. This would also account for the variable and inconsistent occurrence of PH in fetuses presenting the same anatomical defects, due to the probable multifactorial regulation of these endothelial functions.

In conclusion, we demonstrate here that CHDs with right outflow obstruction are a significant risk factor for prenatally acquired PH. A decrease in alveolar multiplication may also be acquired postnatally in infants with these CHDs, as a direct result of the decrease in pulmonary blood flow, but our study shows that the occurrence of fetal PH is not correlated with abnormalities of the pulmonary vasculature, suggesting a role for perfusion-independent mechanisms.

## Supporting Information

Figure S1 **Regression model relating LW to BW for each case.**
(TIF)

Figure S2 **Individual LW/BW values.**
(TIF)
