# Supplementary figures and images for: Pulmonary Hypoplasia Associated with Congenital Heart Diseases: A Fetal Study
Source: PLoS One. 2014 Apr 3;9(4):e93557. doi: 10.1371/journal.pone.0093557 (PMC3974773; doi:10.1371/journal.pone.0093557)

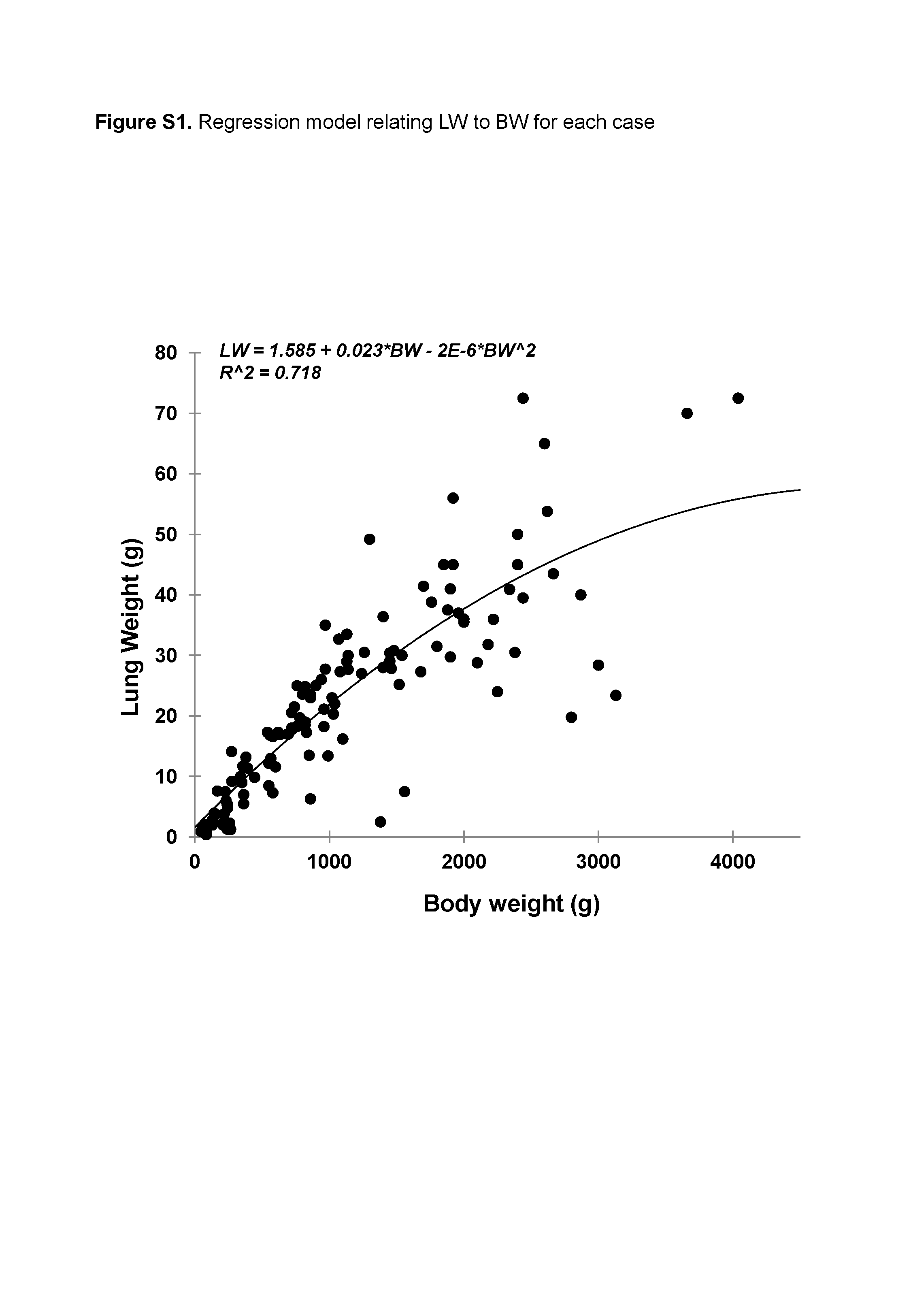

Supplement: Figure S1 — Regression model relating LW to BW for each case. (TIF) [file pone.0093557.s001.tif]

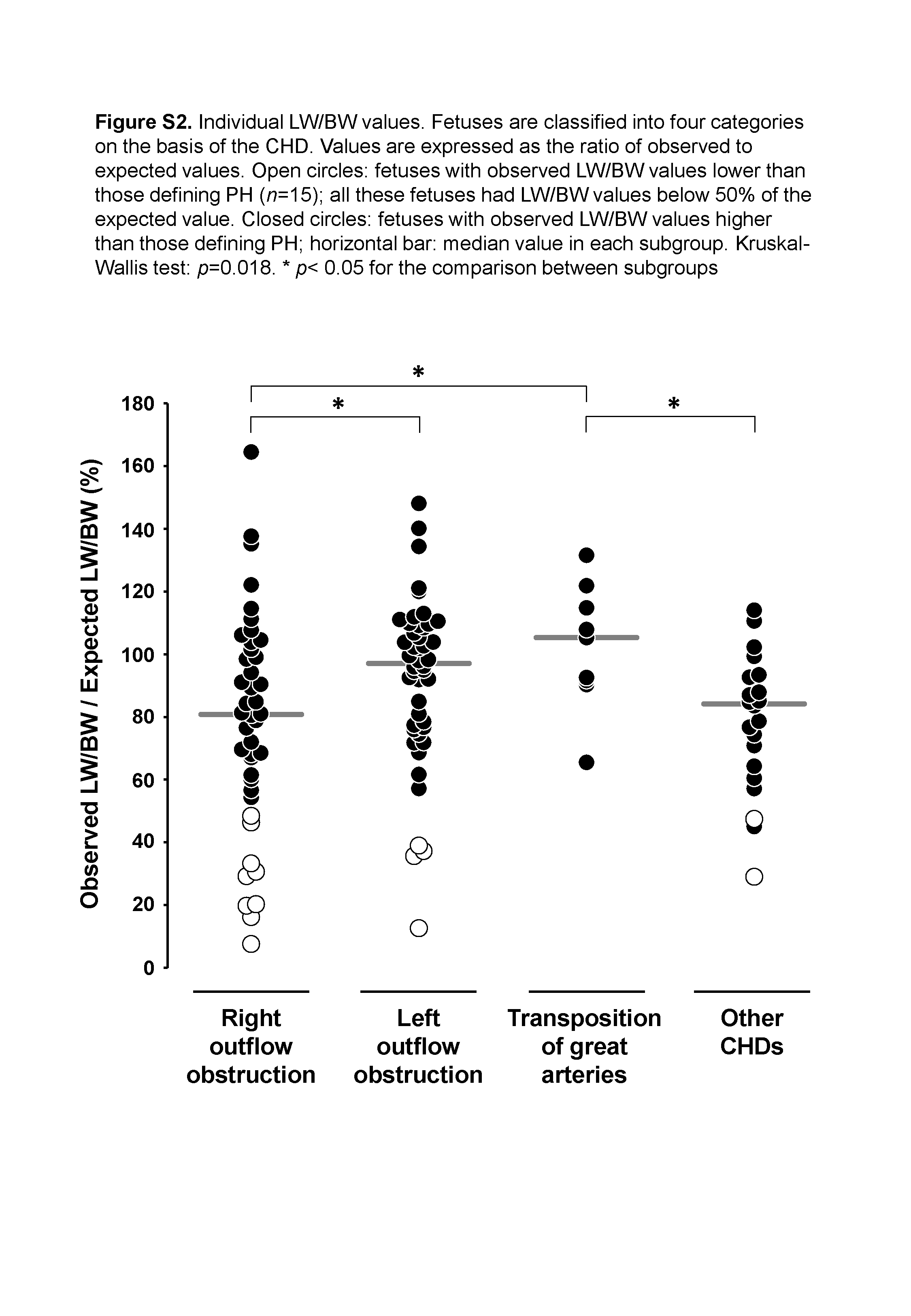

Supplement: Figure S2 — Individual LW/BW values. (TIF) [file pone.0093557.s002.tif]
